# Morphophysiological and biochemical responses of two grape cultivars to phosphorus deficiency and toxicity

**DOI:** 10.1371/journal.pone.0361111

**Published:** 2026-10-26

**Authors:** Maryam Zaeri Behrooz, Rouhollah Karimi, Ali Khadivi, Yazgan Tunç, Lalu Prasad Yadav

**Affiliations:** 1 Department of Horticulture and Landscape Engineering, Faculty of Agriculture, Malayer University, Malayer, Iran; 2 Grape Production and Genetic Improvement Department, Iranian Grape and Raisin Institute, Malayer University, Malayer, Iran; 3 Department of Horticultural Sciences, Faculty of Agriculture and Natural Resources, Arak University, Arak, Iran; 4 Republic of Türkiye, Ministry of Agriculture and Forestry, General Directorate of Agricultural Research and Policies, Hatay Olive Research Institute Directorate, Hassa, Hatay, Türkiye; 5 ICAR - Central Institute for Arid Horticulture, Bikaner, Rajasthan, India; Nuclear Science and Technology Research Institute, IRAN, ISLAMIC REPUBLIC OF

## Abstract

Phosphorus (P) is a vital macronutrient for grapevine (*Vitis vinifera* L.); however, the physiological and biochemical mechanisms governing grapevine adaptation across varying P supply levels—from deficiency to toxicity—require further clarification. This study evaluated the morphophysiological, nutritional, and biochemical adaptations of two contrasting grapevine cultivars, ‘Khalili’ and ‘Perlette’, to varying P levels: 0 mM (deficiency), 2.5 mM (sufficiency), and 5 mM (excess/toxicity). Results demonstrated that vegetative growth parameters—including stem length, stem diameter, lateral shoot number, and shoot dry weight—peaked under P sufficiency (2.5 mM), whereas severe P deficiency and P excess significantly impaired shoot development. In contrast, root dry weight remained comparable between 2.5 and 5 mM P, resulting in a higher root-to-shoot ratio under P excess. Nutrient profiling revealed distinct tissue-specific partitioning: P supply promoted foliar P and N accumulation while altering root macronutrient status (N, P, K, Ca, Mg), but sharply reduced foliar Fe and Zn concentrations. In roots, elevated P suppressed Fe, Mn, and Cu accumulation while enhancing Zn uptake. Both P deficiency and excess triggered oxidative stress, evidenced by up-regulated activities of antioxidant enzymes (catalase, guaiacol peroxidase, and ascorbate peroxidase) in leaves and roots. Cultivar-specific responses were prominent: P deficiency induced substantial anthocyanin accumulation and higher leaf starch content in ‘Khalili’, whereas ‘Perlette’ maintained more stable ionic homeostasis under P excess. Overall, 2.5 mM P represents the physiological optimum for grapevine vegetative development, while both extreme deficiency and excess impair growth via distinct mineral imbalances and oxidative stress cascades.

## 1. Introduction

Phosphorus is an essential nutrient for plant growth; however, evidence suggests that agricultural soils globally are experiencing a progressive depletion of P reserves. This decline is primarily driven by insufficient P inputs relative to crop removal and soil erosion, with more pronounced depletion observed in regions such as Africa, South America, and Eastern Europe. Moreover, the low mobility of P in soil leads to its rapid fixation by iron (Fe) and aluminum oxides in acidic soils, and by calcium (Ca) compounds in calcareous soils, thereby limiting its availability to plants. Consequently, P deficiency remains a critical constraint to the sustainability of agricultural production globally [1,2]. Nevertheless, P exhibits high mobility within the plant and is redistributed among tissues depending on internal demand and soil P supply [3,4]. P is absorbed primarily as orthophosphate ions (H_2_PO_4_^−^ and HPO_4_^2−^) via inorganic phosphate (Pi) transporters located in the plasma membrane of root cells. P exists in plants in various forms, including inorganic P and simple phosphate esters consisting of a phosphate group esterified to a carbon chain [5]. It can also form pyrophosphate bonds with other phosphate groups, generating high-energy linkages essential for cellular metabolism. The ratio between free inorganic P and organic phosphate esters depends on the total P concentration in the cell. Inorganic P is the metabolically active form, located either in the cytoplasm or stored in the vacuole, while phosphate esters are found in structural forms such as ATP, DNA, phosphorylated metabolites, and phospholipids [6]. Plants exhibit distinct responses in roots and shoots to varying P levels. To maintain nutritional balance, they employ a range of morphological, physiological, and biochemical mechanisms. However, excessive P accumulation can result in toxicity, leading to reduced root growth, morphological abnormalities in roots and shoots, impaired micronutrient uptake, and disruption of physiological functions [7]. Plant responses to P excess are species- and genotype-dependent. Studies in annual crops, including maize, common bean, and barley, have reported genotype-specific effects of high P supply on root growth, tissue P accumulation, and micronutrient balance [8,9]. In the horticultural species Lantana camara, high P supply increased tissue P accumulation and reduced the root-to-shoot ratio without significantly affecting overall growth or flowering, indicating relatively high tolerance to P excess [10]. Excessive P can also disrupt Zn (zinc) nutrition in crops such as maize and potato, with the severity of toxicity depending on plant species, P and Zn concentrations, and growth conditions [11,12]. These findings emphasize that responses to P excess cannot be generalized across species and highlight the need for crop-specific studies in perennial horticultural plants. Consequently, establishing species- and genotype-specific P thresholds is crucial for preventing toxicity and maintaining root health and overall plant performance. In contrast, P deficiency is also a major constraint on plant growth and development. This deficiency can limit shoot, root, and reproductive development and reduce the uptake efficiency of other nutrients. In maize, genotypes with greater lateral root branching density exhibit superior P acquisition, shoot biomass, and grain yield under low-P soil conditions [13]. Morphologically, P deficiency induces root deformation, increases the number of lateral and fine roots, widens the angle between primary and lateral roots, alters the root-to-shoot ratio, and affects symbiotic relationships with mycorrhizae [14]. Physiologically, plants respond to P deficiency through increased phosphatase activity, secretion of organic acids, and changes in the expression of phosphate transporters. These mechanisms facilitate the movement of immobilized P and enhance its availability to roots. At the cellular level, P deficiency leads to increased starch accumulation and replacement of membrane phospholipids with sulfolipids to maintain photosynthetic activity [15]. Soil application of P, particularly at depths of 20–40 cm, increased the yield of ‘Pinot Noir’ grapes [16]. Research on rice has shown that modern varieties, by optimizing root architecture and directing P to sensitive organs, can mitigate the adverse effects of P deficiency. Precise nutrient management is therefore crucial for sustaining crop performance [17]. Experiments on 11 rice genotypes indicated that increased P improved plant height, tiller number, leaf area, and dry matter accumulation. Notable genotypic differences persisted, with some genotypes performing better under P-deficient conditions [18]. In *Phaseolus vulgaris*, P deficiency reduces root nodule formation and limits N fixation, accompanied by alterations in the expression of related genes [19]. Beyond its direct effects on plants, inefficient P management can lead to serious environmental impacts in agricultural soils, including eutrophication, accumulation of heavy metals, and risks associated with radioactive phosphogypsum [20,21]. Therefore, the monitoring and sustainable management of P in agricultural and horticultural systems—which require a precise understanding of its role in plant nutrition and the design of strategies to ensure an adequate supply—are of great importance. This approach necessitates determining the P requirements of each plant species and considering changes in these requirements throughout different growth stages to align P supply with actual plant demand. Despite substantial advances in P research in viticulture, significant knowledge gaps persist regarding the interactive effects of P deficiency and excess on the morphophysiology, nutrient status, and biochemical traits of grapevine leaves and roots. For example, Stefanello et al. [22] evaluated critical P thresholds in vineyard soils and leaves, and Gautier et al. [23] demonstrated that scion and rootstock genotypes exert profound, differential controls over physiological and transcriptional responses to low phosphate availability in grapevine. Similarly, Zhao et al. [24] examined morphophysiological responses of hydroponically grown grapevines under P deficiency. However, a comprehensive comparative investigation assessing how different grapevine cultivars adapt simultaneously to the dual extremes of P stress—deficiency and excess—remains lacking, particularly regarding belowground and aboveground antioxidant defense mechanisms and metabolic adjustments. Addressing this gap is particularly important because inappropriate P management, including its over-application, may adversely affect vine growth, nutrient balance, and physiological performance. Therefore, the present study systematically investigates the morphophysiological, nutritional, and biochemical responses of two contrasting grapevine cultivars, ‘Khalili’ and ‘Perlette’, across a wide gradient of P availability, including deficiency, sufficiency, and excess. The distinct contribution of this study lies in its integrated comparative assessment of these two cultivars across a broad spectrum of morphological, physiological, nutritional, antioxidant, and biochemical traits. Specifically, the primary objectives were to: (i) elucidate the morphophysiological, enzymatic antioxidant, and metabolic mechanisms governing grapevine responses across the full gradient of P availability (from severe deficiency to induced toxicity); and (ii) identify genotype-specific physiological thresholds and adaptive strategies distinguishing ‘Khalili’ from ‘Perlette’ under contrasting P regimes.

## 2. Materials and methods

### 2.1. Plant material and treatments

Grapevine cultivars ‘Khalili’ and ‘Perlette’ were obtained from the Grape and Raisin Research Garden, Malayer Research Institute, Iran. After rooting, uniform cuttings were transplanted into 8-L pots filled with a perlite-based growth medium. The experiment was conducted under greenhouse conditions as a 2 × 3 factorial arrangement in a completely randomized design, with three biological replicates per treatment. Each replicate consisted of three pots, with one plant per pot. For 100 days, plants were irrigated three times per week with a modified Hoagland nutrient solution containing 5 mM KNO_3_, 4 mM Ca(NO_3_), 1 mM NH_4_NO_3_, 2 mM MgSO_4_, 0.1 mM FeSO_4_, 0.1 mM Na_2_EDTA, 0.1 mM H_3_BO_3_, 0.13 mM MnSO_4_, 0.03 mM ZnSO_4_, 1 mM Na_2_MoO_4_, and 0.1 mM CuSO_4_. P treatments were established by supplying KH_2_PO_4_ at concentrations of 0 mM (P-deficient), 2.5 mM (P-sufficient), and 5 mM (high-P). The KCl concentration was adjusted accordingly to maintain the same total potassium (K) concentration across all treatments. To minimize salt accumulation in the growth medium, the pots were leached weekly with distilled water. The pH and electrical conductivity (EC) of the nutrient solution were monitored twice weekly. Plants were inspected regularly throughout the experiment for pest infestation and disease symptoms. Photographs of the two grape cultivars under the different P treatments are shown in Fig 1.

**Fig 1:**
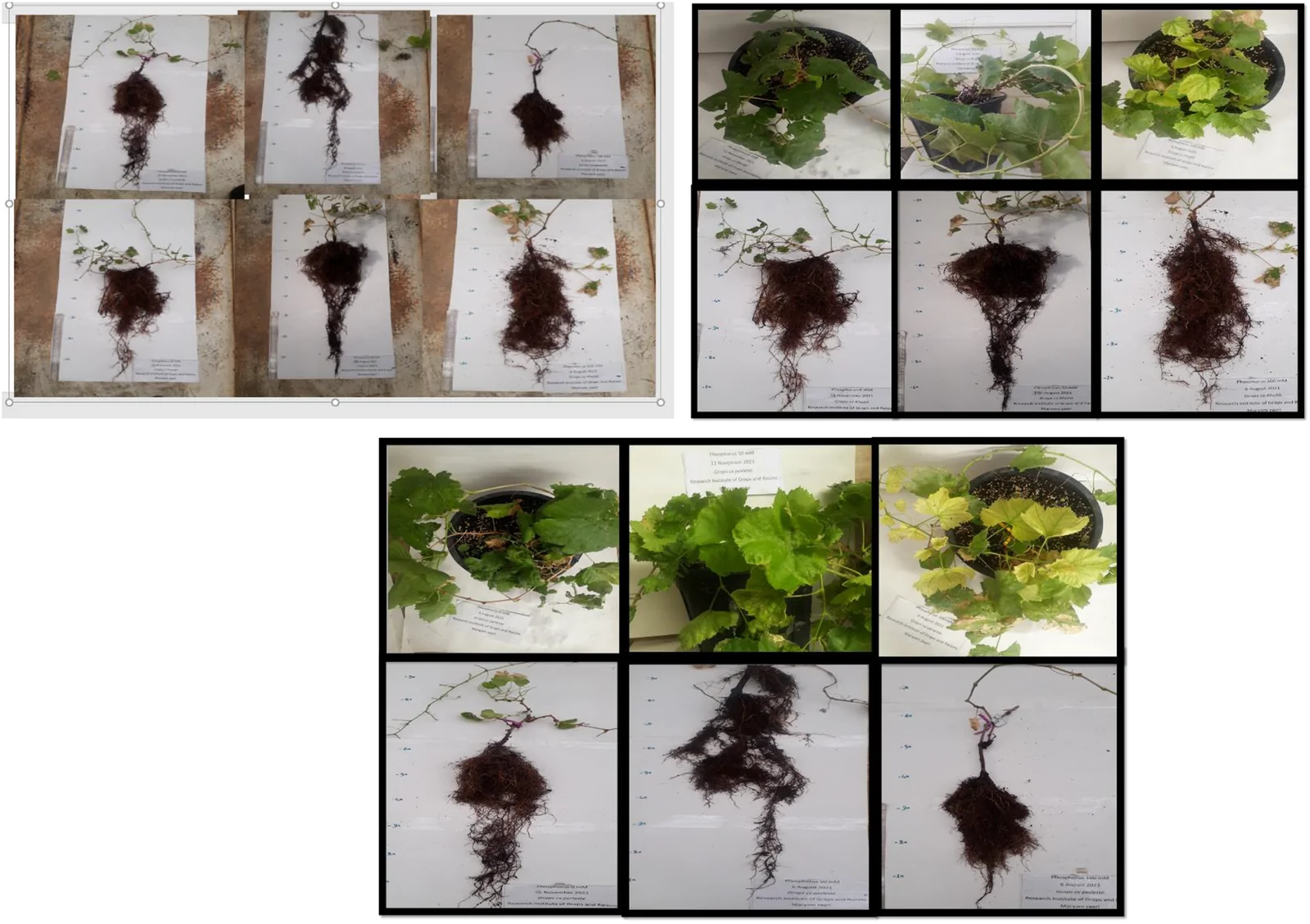
Photographs of the two grape cultivars under the different P treatments (‘Khalili’ and ‘Perlette’). These photographs were taken as part of the present study and are provided by the first author, Maryam Zaeri Behrooz, and are free from any third-party copyright restrictions.

#### 2.1.1. Statement specifying permissions.

For this study, we acquired permission to study grape issued by the Agricultural and Natural Resources Ministry of Iran.

#### 2.1.2. Statement on experimental research and field studies on plants.

The cultivated or wild-growing plants sampled comply with relevant institutional, national, and international guidelines and domestic legislation of Iran. Inclusivity-in-global-research-questionnaire is shown in S1 File.

### 2.2. Morphometric indices

At the end of the 14th week, stem length, stem diameter, number of lateral stems, leaf number, root length, stem fresh weight (FW) and dry weight (DW), root FW and DW, and the root-to-shoot ratio were recorded. Leaf number and the number of lateral stems were counted, whereas stem length and stem diameter were measured on intact plants using a graduated ruler and a digital caliper (Mitutoyo CD-6, Mitutoyo Corporation, Japan), respectively. The vines were then removed from the pots. Root length was measured using a graduated ruler. Root FW and DW, as well as stem FW and DW, were determined using a digital balance (Scout SPX223, Ohaus Corporation, USA), after which the samples were oven-dried at 70 °C for 72 h to determine their DW.

### 2.3. Total chlorophyll

Total chlorophyll (ChL) content was determined by extracting fresh leaf tissue with 80% (v/v) aqueous acetone. The absorbance of the extracts was measured at 663, 645, and 652 nm using a UV–visible spectrophotometer (Lambda 25, PerkinElmer, USA). Chlorophyll a, chlorophyll b, and ChL contents were calculated using the equations of Arnon [25] and expressed as mg g ^−1^ fresh weight (FW).

### 2.4. Total soluble protein

The measurement of soluble protein content was performed according to the Bradford method, in which 0.5 g of leaf tissue was ground in a porcelain mortar in the presence of 5 mL of extraction buffer containing 1 mM Tris (pH 7.0) and then centrifuged at 6,000 × g for 20 min. Subsequently, 100 μL of the supernatant was mixed with 5 mL of Bradford reagent (10% glacial acetic acid + 25% ethanol + 65% distilled water + 0.1% (v/w) Coomassie Brilliant Blue G 250 solution) and the absorbance of the resulting mixture was measured at 595 nm using a spectrophotometer. A standard curve was prepared from different concentrations of bovine albumin and used to determine the concentration of soluble proteins (expressed as mg g^-1^ FW).

### 2.5. Antioxidant enzyme activities

To determine antioxidant enzyme activities, leaf samples were powdered in liquid nitrogen. Subsequently, 100 mg of the powdered tissue was homogenized in 1.0 mL of 50 mM sodium phosphate buffer (pH 7.8) containing 1 mM EDTA and 2% (w/v) polyvinylpolypyrrolidone (PVPP). The homogenates were centrifuged at 10,000 × g for 20 min at 4 °C. The resulting supernatants were collected and used as crude enzyme extracts. Catalase (CAT) activity was determined by monitoring the decrease in absorbance at 240 nm resulting from the decomposition of hydrogen peroxide (H_2_O_2_), according to Bergmeyer [26]. The reaction mixture had a final volume of 3.0 mL and contained 50 mM sodium phosphate buffer (pH 7.0), 1.0 mM EDTA, 50 µL of crude enzyme extract, and H_2_O_2_ at a final concentration of 15 mM. The reaction was initiated by adding H_2_O_2_, and the decrease in absorbance at 240 nm was recorded for 3 min. CAT activity was calculated using the molar extinction coefficient of H_2_O_2_ at 240 nm (ε_240_ = 39.4 M^-1^ cm^-1^). One unit of CAT activity was defined as the amount of enzyme that decomposed 1.0 µmol of H_2_O_2_ per minute under the assay conditions. Guaiacol peroxidase (GPX) activity was determined spectrophotometrically using a modified guaiacol oxidation assay based on the method of Herzog and Fahimi [27], with minor modifications. The assay was performed in a final volume of 3.0 mL containing 1.0 mL of crude leaf enzyme extract, guaiacol at a final concentration of approximately 2.56 mM, H_2_O_2_ at a final concentration of approximately 4.4 mM, and 50 mM sodium phosphate buffer (pH 7.0). The enzyme extract, guaiacol, and buffer were pre-mixed, and the reaction was initiated by adding H_2_O_2_. The increase in absorbance resulting from tetraguaiacol formation was monitored at 470 nm for 3 min. GPX activity was calculated using the molar extinction coefficient of tetraguaiacol at 470 nm (ε_470_ = 26.6 mM ^−1^ cm^−1^). Ascorbate peroxidase (APX) activity was determined by monitoring the decrease in absorbance at 290 nm for 1 min due to ascorbate oxidation, following Nakano and Asada [28]. The reaction mixture had a final volume of 3.0 mL and contained 50 mM sodium phosphate buffer (pH 7.0), 0.5 mM ascorbate, 0.1 mM Na_2_EDTA, 1.2 mM H_2_O_2_, and 100 µL of crude enzyme extract. The reaction was initiated by adding H_2_O_2_. APX activity was calculated using the molar extinction coefficient of ascorbate at 290 nm (ε_290_ = 2.8 mM^−1^ cm^−1^). One unit of APX activity was defined as the amount of enzyme that oxidized 1.0 µmol of ascorbate per minute under the assay conditions. The specific activity of all enzymes was expressed as U mg^−1^ soluble protein.

### 2.6. Starch content

To extract starch, first, 0.5 g of leaf samples were homogenized in 5 mL of ethanol at 80% concentration to separate starch. The mixture was centrifuged at 6,000 × g, and the resulting pellet was washed three additional times with 80% ethanol. The residual sediments were dehydrated, and 5 mL of water, along with 6.5 mL of 52% perchloric acid, were introduced to it. The tubes holding the samples were maintained at 4°C for 15 minutes and subjected to centrifugation once more. After centrifugation, the liquid phase was transferred into a sample vial and maintained on ice. After adding 2.3 mL of perchloric acid to the tubes, the extraction was repeated and centrifuged for the third time. At this stage, the collected liquid phase was separated and adjusted to 100 mL with the addition of distilled water. From this solution, 0.2 mL was drawn and combined with 3 mL of anthrone. The tubes were then immersed in a hot water bath at 90 °C for 10 minutes. Following this, the tubes were rapidly chilled in an ice-water bath, and the absorbance of the samples was measured at 630 nm using a spectrophotometer. Starch concentration was determined based on the standard curve of starch solution and expressed as mg g^−1^ fresh weight [29].

### 2.7. Total anthocyanin

Total anthocyanin content was determined using a modified spectrophotometric procedure based on Giusti and Wrolstad [30]. Fresh leaf tissue (0.1 g) was homogenized in 10 mL of acidified methanol (methanol:HCl, 99:1, v/v) and incubated in the dark for 24 h. The absorbance of the extract was measured at 530 nm using a spectrophotometer. Anthocyanin concentration was calculated using a molar extinction coefficient of 34,300 L mol^−1^ cm^−1^ for cyanidin-3-glucoside in acidified methanol, as attributed to Siegelman and Hendricks [31], and expressed as mg cyanidin-3-glucoside equivalents per g fresh weight.

### 2.8. Nutrient content (in leaves and roots)

Nutrient concentrations in grapevine leaves and roots were determined at the end of the experiment. Mature and healthy leaves and roots were collected, thoroughly washed with distilled water, and oven-dried at 72 °C for 72 h. The dried samples were then ground into a fine powder and used for nutrient analysis.

Total nitrogen (N) concentration was determined using the Kjeldahl method. For the determination of K, magnesium (Mg), Ca, Fe, Zn, manganese (Mn), and copper (Cu), 0.5 g of the powdered plant material was subjected to wet digestion using a modified procedure based on Abdel‐Shafy et al. [32]. In this modification, the volumes of all reagents were reduced by half. Briefly, 5 mL of concentrated nitric acid (65%) was added to each sample, and the samples were incubated in a water bath at 65 °C for 2 h. After cooling, 1.3 mL of H_2_O_2_ (20%) was added to each digest. The digested samples were then filtered through Whatman No. 42 filter paper, and the final volume was adjusted to 25 mL with distilled water. Potassium concentration was measured in the resulting digest using a flame photometer (Model G 405, Germany). The concentrations of Mg, Ca, Fe, Zn, Mn, and Cu were determined using an atomic absorption spectrophotometer (AAnalyst 70, PerkinElmer, USA).

P concentration was determined separately using the vanadomolybdophosphoric acid colorimetric method at 470 nm, according to Kalra [33]. For P analysis, the plant material was subjected to the same nitric-acid digestion procedure, except that H_2_O_2_ was not added. Nutrient concentrations were calculated based on the initial sample mass and the final extract volume, with appropriate correction for any additional dilution.

### 2.9. Data analysis

The experimental data (S2 File) were analyzed using the General Linear Model (GLM) procedure in SAS software (version 9.1; SAS Institute Inc., Cary, NC, USA). The effects of cultivar, P treatment, and their interaction were evaluated using an analysis of variance (ANOVA) model. When significant differences were detected, treatment means were separated using Duncan’s multiple range test at the 5% significance level (*p <* 0.05). All results are presented as means ± standard error (SE).

## 3. Results

### 3.1. Morphometric indices

P supply exerted significant effects on all recorded morphometric traits (Table 1). Genotypic differences were observed in stem length, stem diameter, number of lateral stems, and leaf number. Significant Genotype × P concentration interactions were recorded for root length and leaf number, indicating cultivar-specific responses to P availability. Compared with P sufficiency (2.5 mM), P deficiency (0 mM) caused a substantial reduction in all measured traits. Under P toxicity (5 mM), while most vegetative parameters declined, leaf number and the root-to-shoot ratio increased, deviating from the overall trend observed in other growth traits.

**Table 1:** The effect of different concentrations of phosphorus (P) on morphological traits of two grape cultivars (‘Khalili’ and ‘Perlette’).

Treatment	Stem length (cm)	Root length (cm)	Stem dry weight (g)	Root dry weight (g)	Root-shoot ratio	Number sub-branches	Stem diameter (cm)	Number of leaves
Khalili	38.1 ± 1.47 b	59.1 ± 1.3 a	13 ± 0.36 a	22 ± 0.66 a	1.80 ± 0.07 a	3 ± 0.15 b	1.17 ± 0.07 a	18.4 ± 0.91 b
Perlette	48.4 ± 1.46 a	58.7 ± 0.4 a	12.7 ± 0.34 a	21.4 ± 0.65 a	1.79 ± 0.06 a	4 ± 0.25 a	1.05 ± 0.06 b	23.7 ± 0.19 a
P_0_	20.6 ± 0.88c	44.5 ± 0.83 c	10.6 ± 0.17 b	15.1 ± 0.26 b	1.45 ± 0.03 b	2 ± 0.09 b	0.94 ± 0.05 b	10.1 ± 0.23 c
P_2.5_	60.8 ± 1.04 a	75.1 ± 1.5 a	16.1 ± 0.28 a	25.8 ± 0.71 a	1.53 ± 0.05 b	6 ± 0.32 a	1.2 ± 0.05 a	21.6 ± 0.58 b
P_5_	37.3 ± 1.62 b	50.8 ± 1.2 b	10.5 ± 0.31 b	24.1 ± 0.62 a	2.04 ± 0.11 a	3 ± 0.14 b	0.78 ± 0.03 c	31.6 ± 0.81 a
Perlette × P_0_	25 ± 1.6 cd	43.3 ± 1.03 d	11.5 ± 0.33b	15 ± 0.33 b	1.37 ± 0.19 b	2 ± 0.19 c	0.93 ± 0.03 d	11.1 ± 0.33 d
Perlette × P_2.5_	75 ± 1.0 a	80.3 ± 1.1 a	16.1 ± 0.88 a	25.3 ± 1.5 a	1.62 ± 0.15 ab	7 ± 0.33 a	1.12 ± 0.04 b	24.6 ± 0.50 b
Perlette × P_5_	45.3 ± 1.8 b	45.6 ± 1.01 d	10.3 ± 0.50 b	24.1 ± 1.5 a	2.30 ± 0.23 a	4 ± 0.0 bc	0.73 ± 0.03 e	35.6 ± 0.50 a
Khalili × P_0_	18.3 ± 1.17 d	45.3 ± 1.8 d	10.1 ± 0.33 b	15.3 ± 0.76 b	1.54 ± 0.08 ab	2 ± 0.19 c	1 ± 0.05 c	9.1 ± 0.33 d
Khalili × P_2.5_	66.1 ± 2 a	73 ± 1.4 b	16.3 ± 0.19 a	26.3 ± 1.57 a	1.41 ± 0.09 b	4 ± 0.69 b	1.23 ± 0.04 a	18.6 ± 0.38 c
Khalili × P_5_	29.3 ± 1.3 c	57.6 ± 0.83 c	10.6 ± 0.83 b	24.3 ± 1.26 a	2.41 ± 0.28 a	2 ± 0.19 c	0.83 ± 0.03 d	29.6 ± 0.01b

Means with common letters in each column are not significantly different (Duncan’s multiple range test, *p ≤* 0.01). Data are means of three replications.

Regarding cultivar performance, ‘Khalili’ exhibited superior stem length and diameter, whereas ‘Perlette’ produced a higher number of lateral stems and leaves. Analysis of the Genotype × P interaction revealed that ‘Perlette’ achieved its maximum root length at 2.5 mM P, but the minimum at 5 mM P. Furthermore, ‘Perlette’ showed the highest leaf count under P toxicity, whereas the lowest was recorded under P deficiency (Table 1).

### 3.2. Macronutrients (leaf and root)

P treatments significantly affected all measured macronutrients in both leaves and roots, including leaf K. The cultivar factor significantly affected only leaf N and Ca, and the interaction between cultivar and P concentration was not significant for any leaf or root macronutrient. Leaf N content differed significantly between the two cultivars, with ‘Khalili’ exhibiting higher leaf N concentration than ‘Perlette’. In leaves, the highest N content was observed under sufficient and high P treatments (2.5 and 5 mM), whereas the lowest N content occurred under P deficiency (0 mM). In roots, N content increased significantly with P application from 0 to 2.5 mM. Although N content slightly decreased at 5 mM, this reduction was not statistically significant and remained higher than in the P-deficient treatment. In leaves, K content was higher under both P deficiency and toxicity than under sufficient P, and cultivar had no significant effect on leaf or root K. In roots, K content increased significantly with increasing P. P content was similar in both cultivars, and increasing P from 0 to 2.5 mM and then to 5 mM significantly increased P content in both leaves and roots. In leaves, the ‘Khalili’ cultivar exhibited higher Ca content than ‘Perlette’. Leaf Ca content remained unchanged under P deficiency but decreased under P toxicity, whereas in roots, Ca content increased significantly with P application (2.5 and 5 mM) compared to the P-deficient control. Magnesium content was similar in both cultivars. In leaves, Mg decreased significantly when P increased from 2.5 to 5 mM, while no change was observed under P deficiency. In roots, the highest Mg content was observed at 2.5 mMP, which was significantly higher than under P deficiency (0 mM), but decreased at 5 mMP (Table 2).

**Table 2:** The effect of different concentrations of phosphorus (P) on contents of root and leaf macronutrients of two grape cultivars (‘Khalili’ and ‘Perlette’).

Treatment	N (%)	K (%)	P (%)	Ca (%)	Mg (%)
**Leaf elements**					
Khalili	2.78 ± 0.68 a	1.71 ± 0.20 a	0.180 ± 0.058 a	2.77 ± 0.76 a	0.21 ± 0.067 a
Perlette	2.02 ± 0.69 b	1.73 ± 0.31 a	0.104 ± 0.059 a	1.98 ± 0.59 b	0.19 ± 0.06 a
P_0_	1.76 ± 1.01 b	1.91 ± 0.25 b	0.046 ± 0.021 c	2.68 ± 0.87 a	0.23 ± 0.063 a
P_2.5_	2.51 ± 0.72 a	1.58 ± 0.4 c	0.120 ± 0.024 b	2.86 ± 0.19 a	0.25 ± 0.062 a
P_5_	2.83 ± 1.01 a	2.18 ± 0.30 a	0.150 ± 0.024 a	1.7 ± 0.82 b	0.13 ± 0.02 b
**Root elements**					
P_0_	2.71 ± 0.84 b	1.76 ± 0.37 b	0.043 ± 0.045 b	0.58 ± 0.16 b	0.135 ± 0.03 b
P_2.5_	3.33 ± 0.65 a	2.25 ± 0.45 a	0.063 ± 0.018 a	0.85 ± 0.31 a	0.175 ± 0.03 a
P_5_	3.12 ± 0.60 ab	2.31 ± 0.57 a	0.072 ± 0.050 a	0.86 ± 0.25 a	0.055 ± 0.04 ab

Means with common letters in each column are not significantly different (Duncan’s multiple range test, p ≤ 0.01). Data are means of three replications.

### 3.3. Micronutrients (leaf and root)

P concentration significantly affected the concentrations of leaf Fe, Zn, and Cu, as well as all root micronutrients (Fe, Mn, Zn, and Cu) at the 1% probability level (p≤0.01). However, neither the cultivar factor nor the cultivar ×P interaction had a significant effect on any of the measured micronutrients in leaves. In leaves, increasing P concentration significantly decreased Fe and Zn contents, whereas Cu concentration was significantly higher under sufficient and high P levels (2.5 and 5 mM) compared to the P-deficient control (0 mM). Leaf Mn content was not significantly affected by P treatments. In roots, increasing P supply resulted in significant reductions in Fe, Mn, and Cu contents, whereas root Zn content significantly increased with increasing P concentration up to 5 mM (Table 3).

**Table 3:** The effect of cultivar and phosphorus treatment on grape root and leaf micronutrients. Means ± SE (N = 3).

Treatment	Fe (ppm)	Mn (ppm)	Zn (ppm)	Cu (ppm)
**Leaf elements**				
Khalili	33 ± 0.17 a	15.4 ± 0.36 a	16.2 ± 0.59 a	7.6 ± 0.34 a
Perlette	30 ± 0.19 a	14.2 ± 0.25 a	15.6 ± 0.66 a	7.2 ± 0.26 a
P_0_	48 ± 3.01 a	15.1 ± 0.44 a	21 ± 0.23 a	4.1 ± 0.16 b
P_2.5_	30 ± 2.1 b	15.1 ± 0.59 a	17.5 ± 0.31 b	8.6 ± 0.20 a
P_5_	16 ± 1.2 c	14.1 ± 0.41 a	9.1 ± 0.39 c	9.5 ± 0.22 a
**Root elements**				
P_0_	170 ± 6.1 a	67 ± 0.87 a	58.3 ± 1.42 c	12 ± 0.07 a
P_2.5_	130 ± 4.14 b	51 ± 1.22 b	64.3 ± 1.63 ab	10.1 ± 0.28 b
P_5_	110 ± 4.9 c	38 ± 0.76 c	77.8 ± 2.22 a	8.16 ± 0.38 c

Means with common letters in each column are not significantly different (Duncan’s multiple range test, p ≤ 0.01). Data are means of three replications.

### 3.4. Antioxidant enzymes (leaf and root)

P treatment significantly affected the activities of the antioxidant enzymes GPX, CAT, and APX in the leaves and roots of the two grapevine cultivars,‘Perlette’ and ‘Khalili’. For all three enzymes, P deficiency (0 mM) generally resulted in the highest enzyme activity in both tissues and cultivars. Increasing P concentration to 2.5 mM, representing sufficient P conditions, significantly reduced the activities of GPX, CAT, and APX in both leaves and roots. Increasing P concentration to 5 mM partially restored enzyme activity in the roots. In the leaves, the response to 5 mM P depended on the enzyme and cultivar: CAT activity did not increase significantly, whereas APX activity increased significantly in both cultivars. GPX activity also showed a significant response to the increase from 2.5 to 5 mM P, although the direction of change differed between the cultivars. Comparisons between cultivars showed that ‘Perlette’ generally exhibited higher GPX activity under P deficiency, but this superiority was not consistent across all treatments and tissues. For CAT, ‘Khalili’ showed higher activity than ‘Perlette’ under P deficiency and in the roots under toxic P conditions. For APX, ‘Perlette’ had higher activity than ‘Khalili’ in leaves under P deficiency, whereas no significant difference was observed between the cultivars under sufficient or toxic P conditions (Fig 2).

**Fig 2:**
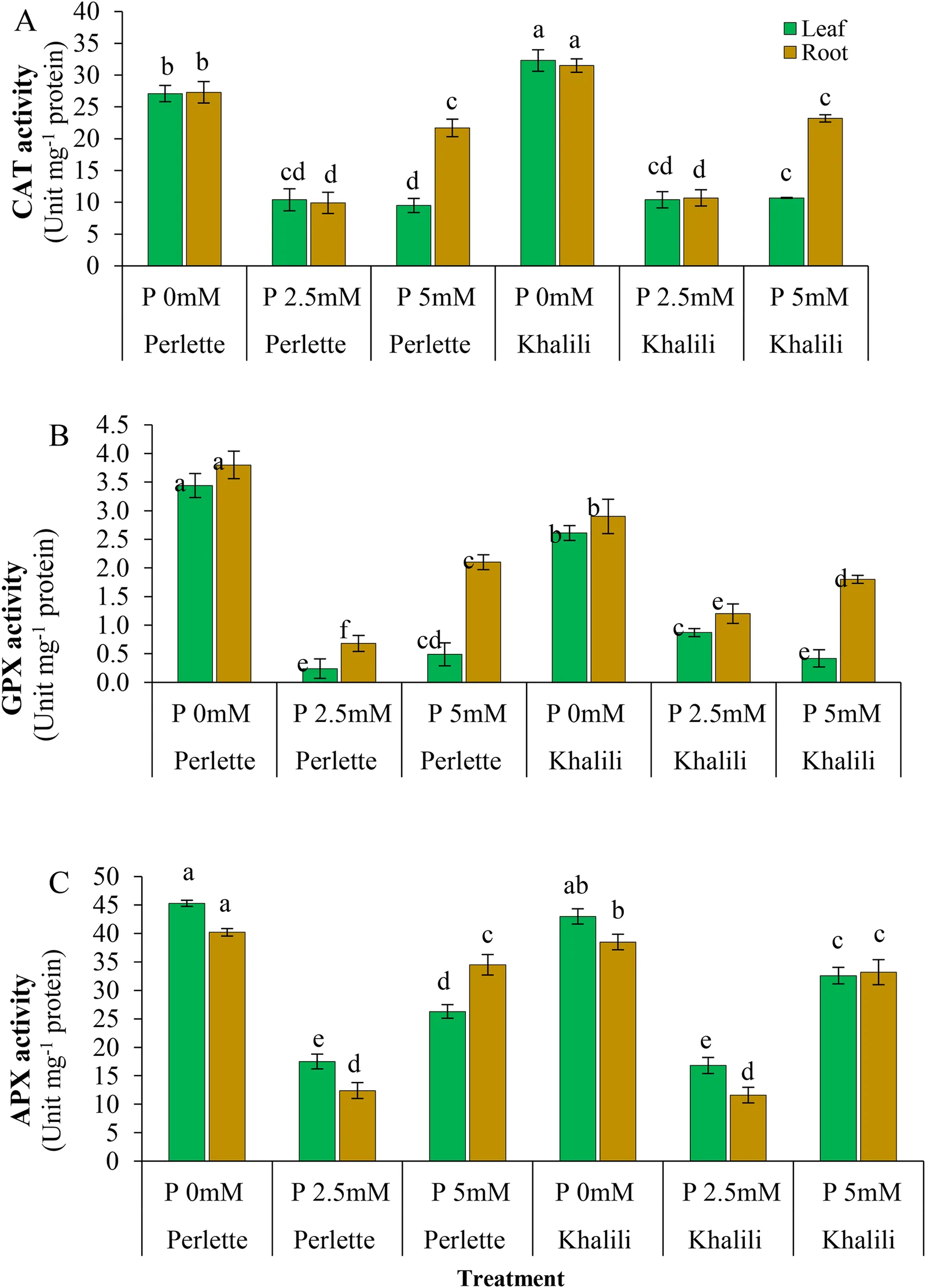
The effect of different concentrations of phosphorus (P) on activities of leaf and root catalase (CAT; panel A), guaiacol peroxidase (GPX; panel B) and ascorbate peroxidase (APX; panel C) of two grape cultivars (‘Khalili’ and ‘Perlette’). Means with common letters in each column are not significantly different (Duncan’s multiple range test, *p* ≤ 0.01). Data are means of three replications.

### 3.5. Leaf starch

The effects of cultivar, P treatments and their interactions on leaf starch content were significant at the 1% probability level. With increasing P concentration from 0 to 5 mM, leaf starch content showed a decreasing trend in both cultivars (Table 4). The greatest amount of leaf starch was associated with vines of the ‘Khalili’ cultivar under P deficiency stress, and the least starch content was found in vines of the ‘Perlette’ cultivar under the 5 mM P treatment (Table 4).

**Table 4:** The effect of different concentrations of phosphorus (P) on leaf starch, protein, anthocyanin, and chlorophyll content of two grape cultivars (‘Khalili’ and ‘Perlette’).

Treatment	Starch (mg g^-1^ FW)	Anthocyanin (mg g^-1^ FW)	Protein (mg g^-1^ FW)	Chlorophyll (mg g^-1^ FW)
Khalili	2.86 ± 0.14 a	1.4 ± 0.127 a	14.2 ± 0.43 a	4.11 ± 0.20 a
Perlette	2.03 ± 0.10 b	1.3 ± 0.129 a	11.3 ± 0.37 b	4.06 ± 0.18 a
P_0_	3.77 ± 0.15 a	2.82 ± 0.04 a	9.3 ± 0.31 c	6.8 ± 0.25 a
P_2.5_	2.13 ± 0.12 b	1.12 ± 0.029 b	16.9 ± 0.41 a	4.04 ± 0.18 b
P_5_	1.43 ± 0.08 b	0.24 ± 0.01 c	11.8 ± 0.38 b	1.26 ± 0.15 c
Perlette × P_0_	3.08 ± 0.15 b	2.85 ± 0.12 a	8.03 ± 0.31 e	6.7 ± 0.16 a
Perlette × P_2.5_	2.01 ± 0.12 c	1.05 ± 0.05 b	15.06 ± 0.56 b	3.8 ± 0.15 c
Perlette × P_5_	1.01 ± 0.08 e	0.23 ± 0.02 c	10.3 ± 0.48 d	1.08 ± 0.10 e
Khalili × P_0_	4.46 ± 0.20 a	2.81 ± 0.01 a	10.6 ± 0.5 cd	6.04 ± 0.21 b
Khalili × P_2.5_	2.26 ± 0.12 c	1.15 ± 0.07 b	18.7 ± 0.5 a	4.1 ± 0.18 c
Khalili × P_5_	1.87 ± 0.10 cd	0.25 ± 0.04 c	13.3 ± 0.69 bc	2.15 ± 0.15 d

Means with common letters in each column are not significantly different (Duncan’s multiple range test, p ≤ 0.01). Data are means of three replications.

### 3.6. Leaf anthocyanins

Leaf anthocyanin content was significantly affected by the interaction between cultivar and P treatments. The trend in leaf anthocyanin content was similar to that observed for leaf starch content. In both cultivars, leaf anthocyanin content was higher under P deficiency stress than under the other P treatments. Conversely, leaf anthocyanin content reached its lowest value under the 5 mM P treatment in both cultivars (Table 4).

### 3.7. Leaf soluble protein

Both cultivar and P concentration significantly affected leaf soluble protein content; however, their interaction (cultivar × P) was not significant. The lowest leaf soluble protein content was observed under P deficiency (0 mM), whereas the highest content occurred under P sufficiency (2.5 mM P). Under P toxicity (5 mM P), leaf soluble protein content decreased compared with that under the P-sufficiency treatment (Table 4).

### 3.8. Leaf total ChL

Leaf ChL content was significantly affected by the interaction between cultivar and P treatments. The trend of ChL changes was similar to that of the protein changes. The level of ChL in the leaves of vines experiencing P deficiency stress was higher in both cultivars compared to other P treatments. In contrast, the content of this photosynthetic pigment reached the lowest value under the 5 mM P treatment in both cultivars (Table 4).

## 4. Discussion

P deficiency substantially affects both plant morphology and physiology, with alterations in root and shoot biomass being among the most prominent responses. During the early stages of P deficiency, primary root elongation may be inhibited, whereas lateral-root development may be stimulated as an adaptive response to enhance P acquisition [6,34].

In the present study, primary-root length was significantly reduced under P deficiency. In both cultivars, root length was greater at 2.5 mM P than at 5 mM P, suggesting that excess P also restricted root elongation. These changes in root growth may involve nutrient- and hormone-signaling networks, including TORC1- and ethylene-related pathways [35]; however, these mechanisms were not directly assessed in the present study. Root dry weight was also significantly lower under P deficiency than under P sufficiency, whereas it did not differ significantly between the P-sufficiency and excess-P treatments. This result is consistent with previous reports that P deficiency can reduce root growth [36–38], although contrasting responses have also been documented [39,40].

These discrepancies may be related to differences in growth stages and P concentrations. At the onset of P deficiency, depending on its severity, root growth may increase while shoot growth decreases or ceases [13]. This stage is temporary, and as the deficiency progresses, root length increases and the root-to-shoot ratio rises to compensate for the enlarged root volume. During this phase, deficiency symptoms in the leaves are less visible. Once this stage passes, root growth declines and eventually ceases [13,41].

In the roots of ‘Perlette’, the formation of cluster-like roots increased at 2.5 mM P but was not observed at 5 mM P. Because the formation of these roots was not investigated mechanistically in the present study, this result should be interpreted as an association rather than direct evidence that high P inhibited their formation [40].

Furthermore, P plays a critical role in the morphology of lateral roots and root branching, affecting not only root growth but also nutrient accessibility. Consequently, plants have developed various strategies to acquire optimal P from the soil, including increased root surface area, specific root length (SRL), and root-to-shoot ratio [42].

P status in plants not only directly influences growth and overall performance but also affects the uptake and distribution of other nutrients. Moreover, the plant genotype can modulate these responses, as the contents of P, N, and K in the leaves of four grape cultivars grafted onto ten different rootstocks have been reported to vary significantly [43].

In the present study, only Ca and N exhibited genotype-dependent differences, with higher concentrations observed in the ‘Khalili’ cultivar compared to ‘Perlette’. The lower leaf N content in ‘Perlette’ may be associated with its higher vegetative growth potential and possible biomass dilution. Although P supply increased the concentrations of several macronutrients, some elements showed non-linear responses to increasing P supply. Leaf Mg decreased at 5 mM P compared with 2.5 mM P, whereas root Mg showed a numerical decrease at 5 mM P after reaching its highest value at 2.5 mM P. P treatments also caused significant changes in micronutrient concentrations. In leaves, increasing P supply significantly decreased Fe and Zn concentrations, whereas Cu concentration was significantly higher at 2.5 and 5 mM P than under P deficiency. Leaf Mn concentration was not significantly affected by P supply. In roots, high P levels induced reductions in Fe, Mn, and Cu and an increase in Zn. This antagonistic decline in foliar Fe and Zn concentrations under elevated P supply is consistent with the coordinated cross-talk mechanisms governing Pi, Zn, and Fe homeostasis [61]. High phosphorus availability suppresses the phosphate starvation response (PSR) mediated by the central transcription factor *PHR1,* which otherwise coordinates the expression of key micronutrient transporters and root-to-shoot translocation pathways. Furthermore, excess Pi disrupts the systemic signaling network that governs micronutrient loading into the xylem (mediated by transporters such as *HMA* and *PHO1* family members), ultimately resulting in restricted foliar allocation of both Zn and Fe [61].

Importantly, Fe plays a key role in lateral root development through the auxin signaling pathway. Fe deficiency suppresses TORC1 pathway activity, leading to inhibited lateral root growth, whereas adequate Fe maintains pathway activation and promotes root development. Conversely, excessive Fe can be detrimental, reducing auxin transport and consequently suppressing root growth [35,44].

In the present study, the highest activities of the antioxidant enzymes CAT, GPX, and APX in both leaves and roots were observed in vines exposed to P deficiency (0 mM P). Antioxidant enzymes play a critical role in mitigating oxidative stress and scavenging reactive oxygen species (ROS). CAT and APX are essential enzymes involved in the regulation and removal of H_2_O_2_. CAT primarily catalyzes the rapid decomposition of H_2_O_2_ under conditions of excessive oxidative stress, whereas APX contributes to the fine regulation of H_2_O_2_ levels during cellular signaling [41]. APX reduces H_2_O_2_ to water using ascorbate as an electron donor. GPX and GPX-like enzymes are important components of the plant antioxidant system and contribute to the reduction of H_2_O_2_ and organic hydroperoxides in various cellular compartments [45]. Increasing the P concentration from 2.5 to 5 mM resulted in a significant recovery of CAT, GPX, and APX activities in the roots. In the leaves, however, the response to 5 mM P was enzyme- and cultivar-dependent. APX activity increased significantly in both cultivars, whereas CAT activity did not show a significant increase. GPX activity increased significantly in ‘Perlette’ but decreased in ‘Khalili’ in response to the increase from 2.5 to 5 mM P. In this context, Fu et al. [46] reported that four days of P deficiency increased peroxidase (POD) and superoxide dismutase (SOD) activities by 89.5% and 51.8%, respectively. The antioxidant defense mechanism is known to be species- and tissue-dependent. For instance, Meng et al. [41] demonstrated that, in Citrus grandis leaves, P deficiency triggered an upregulation of CAT and APX, whereas GPX activity was not significantly modulated. In contrast, opposite responses have been reported in soybean and tea plants, in which CAT activity decreased under P deficiency [47]. Pan et al. [48] reported that the responses of antioxidant enzymes to P deficiency varied among rice cultivars. In sensitive cultivars, SOD, CAT, and POD activities initially increased but subsequently declined with prolonged stress, whereas these activities remained relatively stable in tolerant cultivars. Thus, the effect of P deficiency on CAT activity may depend on cultivar tolerance and stress duration. Therefore, differences among studies may be related to genotype, developmental stage, the severity of P deficiency, and the duration of treatment. More broadly, the effects of mineral nutrient availability on antioxidant enzyme activities have also been reported in grapevine. Karimi et al. [49] showed that different Fe and nitrogen concentrations altered the activities of CAT, GPX, and APX, highlighting the role of mineral nutrition in regulating the plant antioxidant system. In perennial grapevines, sustained investment in antioxidant defense under P stress could reduce the resources available for vegetative growth and reserve accumulation, potentially affecting vine vigor and overwintering capacity. In woody perennials such as grapevine (*Vitis vinifera*), the coordinated stimulation of the enzymatic antioxidant cascade under both deficient (0 mM) and supra-optimal (5 mM) inorganic phosphate (Pi) regimes reveals a distinct, biphasic oxidative pattern governed by nutrient disequilibrium. Recent comparative evidence in the cultivars ‘Khalili’ and ‘Perlette’ elucidates that Pi starvation severely restricts ATP synthesis and photophosphorylation efficiency, whereas Pi excess induces cellular metabolic disturbances; both extremes inevitably trigger uncontrolled leakage of reactive oxygen species (ROS) from chloroplastic electron transport chains and peroxisomal pathways [59]. The marked accumulation of hydrogen peroxide ($H_{\text{2}}O_{\text{2}}$) and membrane lipid peroxidation (quantified via malondialdehyde, MDA) directly accounted for the compensatory induction of protective enzymes, predominantly CAT, GPX, and APX. Nevertheless, the continuous energy demand for maintaining this antioxidant defense creates an exhaustive metabolic sink, deflecting assimilates and ATP away from plant growth and biomass accumulation. Taken together, while previous studies have largely focused on the descriptive phenotypic expression of phosphorus starvation, our findings provide a mechanistic framework by coupling the biphasic enzymatic antioxidant response (CAT, GPX, and APX) with leaf starch and carbon partitioning dynamics. Under nutrient imbalance (both Pi deficiency and excess), grapevines face a physiological trade-off: metabolic energy (ATP) and assimilates are heavily invested into scavenging reactive oxygen species (ROS) and cellular repair mechanisms rather than supporting vegetative biomass and long-term carbohydrate reserve accumulation. In the present study, leaf starch content decreased as P concentration increased from 0 to 5 mM in both cultivars. Leaf starch content was significantly higher in ‘Khalili’ than in ‘Perlette’. Pi plays an important role in the regulation of starch synthesis and partitioning by affecting chloroplast phosphate/triose-phosphate exchange and carbon assimilation and playing a key role in the activity of the special phosphate/triose phosphate transporter in the inner membrane of the chloroplast. This element changes the fixation level of carbon dioxide and the Calvin cycle [50]. Because starch reserves in woody tissues are crucial for spring budburst, the P-deficiency-induced disruption in foliar starch metabolism directly threatens subsequent-season vine productivity.

Leaf soluble protein content was significantly affected by P concentration. It reached its highest value at 2.5 mM P and decreased under 5 mM P compared with the P-sufficiency treatment. This response was consistent with previous reports [51,52].

Protein synthesis can also be influenced by the plant’s P status because phosphorus is a component of important biological molecules, including ATP and nucleic acids [47].

One of the common characteristics of plants under P deficiency is the accumulation of anthocyanins, which is achieved through up‑regulation of genes involved in anthocyanin biosynthesis [53].

The increased leaf anthocyanin content observed under P deficiency in the present study is consistent with previous reports [53,54].

Anthocyanin accumulation plays an important role in ROS scavenging and in protecting chloroplasts against photoinhibition under abiotic stresses, including P and N deficiency [55]. Crucially, in grapevine (*V. vinifera*), phosphorus starvation has been shown to provoke a significant reallocation of secondary metabolites toward anthocyanin synthesis, particularly mono- and acylated anthocyanin derivatives, driven by the coordinated transcriptional upregulation of the key regulatory factor *VvMYBA1* and the downstream structural gene *VvUFGT*. This stress-induced pigmentation is tightly coupled with abscisic acid (ABA) signaling and potentially DELLA-mediated growth restraint pathways, suggesting that anthocyanin hyperaccumulation functions not merely as a visual symptom, but as an active photoprotective and antioxidant adaptation mechanism to cope with energy starvation under severe phosphorus deprivation [60].

Under P deficiency, chlorophyll content per unit leaf mass may increase because leaf expansion and cell enlargement are more strongly restricted than chlorophyll retention. Consequently, the plant reduces the photosynthetic efficiency per unit of ChL to adapt to these conditions [56]. Under P deficiency conditions, the xanthophyll concentration within the leaves increases together with the activity of the xanthophyll cycles and the water cycle, but the efficiency of energy absorption by the photosystem decreases, to eliminate the excess energy and maintain the energy balance of the photons received by ChL and chloroplast [57]. In this study, leaf ChL content was highest under P deficiency and decreased at 2.5 and 5 mM P, reaching its lowest value at 5 mM P in both cultivars. Our study demonstrated that the effect of P on ChL content and leaf growth in grapevine is complex, depending on the interaction between leaf number, leaf area, and ChL biosynthesis. In the P-free treatment (P_0_), leaf ChL content remained relatively high, yet overall plant growth was almost completely halted and the number of leaves was very low; leaves remained dark green and viable, indicating that ChL in existing leaves can be maintained independently of new growth. At moderate P levels (P2.5), plant growth improved, although leaf ChL content was lower than under P deficiency. At high P concentration (P5), leaf ChL content decreased, while leaf number increased and leaf area decreased; nevertheless, overall plant growth continued. These findings emphasize that leaf ChL content alone is not a sufficient indicator of plant health or performance and should be interpreted in conjunction with growth and leaf development parameters.

This finding was consistent with some studies, but differed from others [58]. Maple tree seedlings (*Acer mono* L.) treated with 8 g of P fertilizer indicated a considerable reduction in ChL and carotenoids [42]. The decline in ChL concentration in the leaves of vines fed with high concentrations of P can be due to the oxidative stress resulting from the buildup of P and peroxidation of thylakoid membrane lipids [45]. These pigment dynamics illustrate that grapevines modify their leaf biochemical profile under P stress to balance light absorption with restricted vegetative growth.

## 5. Conclusion

In the present study, the physiological, morphological, and biochemical responses of two grapevine cultivars, ‘Khalili’ and ‘Perlette’, were evaluated under three phosphorus regimes: P deficiency (0 mM), P sufficiency (2.5 mM), and P excess (5 mM). The findings indicate that P sufficiency optimized vegetative growth, biomass accumulation, and soluble protein content, whereas both P deficiency and P excess suppressed vine vigor and disrupted mineral nutrient homeostasis. Cultivars showed distinct adaptive responses: ‘Khalili’ maintained greater shoot elongation and main stem diameter, whereas ‘Perlette’ exhibited a greater capacity for lateral branching and leaf production.

Mechanistically, the extreme phosphorus regimes (0 and 5 mM P) triggered evident oxidative stress, corroborated by the coordinated up-regulation of antioxidant enzymes (CAT, GPX, and APX). Under P deficiency, plants diverted metabolic resources toward anthocyanin accumulation and starch preservation, revealing a physiological trade-off where energy was partitioned from growth toward cell protection. Furthermore, excess P supply antagonistically reduced leaf Fe and Zn concentrations, highlighting systemic signaling crosstalk in micronutrient xylem loading.

This investigation was conducted under controlled greenhouse conditions over 100 days—a timeframe determined by vine viability under the severe nutritional constraints of P deficiency and P excess. Establishing these baseline physiological and nutritional profiles under controlled conditions provides an essential mechanistic framework for interpreting cultivar-specific phosphorus use efficiency before long-term vineyard evaluations.

## Supporting information

S1 File Inclusivity-in-global-research-questionnaire. (DOCX)

S2 File Data of the effect of different concentrations of phosphorus (P) on different traits of two grape cultivars (‘Khalili’ and ‘Perlette’). (XLSX)
