# Supplementary material for: Morphophysiological and biochemical responses of two grape cultivars to phosphorus deficiency and toxicity
Source: PLoS One. 2026 Oct 26;21(10):e0361111. doi: 10.1371/journal.pone.0361111 (PMC13704810; doi:10.1371/journal.pone.0361111)
Supplement: Supplementary file 2 [file pone.0361111.s004.docx]

**Dear Handling Editor,**

We thank you and all reviewers for their careful reading of the manuscript and for the constructive suggestions, which have substantially improved the clarity, rigor, and presentation of the work. Below we respond to each comment point by point.

Regards

**Response to Reviewer 1**

We sincerely thank the reviewer for the careful reading of our manuscript and for the constructive comments and suggestions, which have helped us improve the quality and clarity of the paper. We have revised the manuscript thoroughly in response to all concerns raised. Our point-by-point responses are provided below.

***Comment 1:*** *The manuscript contains numerous grammatical errors, spelling mistakes, and unnatural expressions.*

**Response:** We have carefully revised the entire manuscript to improve language quality, grammar, spelling, and style. All typographical errors and unnatural expressions, including specific errors such as “degra70dation,” have been corrected throughout the text.

***Comment 2:*** *Some references appear questionable. Please check and update all references.*

**Response:** All references were carefully checked, verified, and updated where necessary to ensure accuracy, consistency, and relevance to the study.

***Comment 3:*** *The research gap and novelty should be stated more clearly.*

**Response:** The Introduction section was revised to more clearly delineate the research gap and highlight the novel aspects and significance of this study.

***Comment 4:*** *The Discussion section is too descriptive and lacks deep mechanistic interpretation.*

**Response:** The Discussion section was revised to reduce descriptive content and strengthen the mechanistic interpretation of the findings.

***Comment 5:*** *The results on enzymes, starch, and anthocyanins are interesting but should be better linked to grape physiology.*

**Response:** We have revised the Discussion to better relate the findings on enzyme activity, starch, and anthocyanins to grape physiology and stress responses.

***Comment 6:*** *The limitations of the study should be acknowledged, including the short-term greenhouse experiment and the lack of yield and fruit quality assessments.*

**Response:** We sincerely appreciate the reviewer’s insightful comment. We addressed this point in the revised manuscript by explicitly contextualizing the experimental constraints and the methodological rationale of the study. Specifically, the 100-day experimental duration was governed by plant physiological viability under severe nutrient stress, as prolonging the trial under complete P deficiency (0 mM) and extreme P toxicity (5 mM) would have compromised vine survival and confounded the physiological assays. Furthermore, establishing baseline physiological, morphological, and biochemical indices under strictly controlled greenhouse conditions represented a scientifically indispensable preliminary phase. Given that field environments introduced confounding factors—such as heterogeneous soil matrices, fluctuating moisture regimes, and microclimatic variability—that obscured the direct nutritional effects of the treatments, obtaining robust baseline parameters served as an essential prerequisite to provide a reliable reference framework prior to long-term vineyard evaluations.

Regards

**Response to Reviewer 2**

We sincerely appreciate your positive assessment of our work and your thoughtful feedback. We are pleased that you found our study design and the **assessment** of biochemical and morphological traits to be strengths of the manuscript. We have addressed your specific suggestions to improve clarity, precision, and conciseness as follows.

***Comment 1 (Materials and Methods):*** *Clarification of the number of plants represented by each replicate would improve reproducibility.*

**Response:** We have revised the Materials and Methods section to explicitly state the number of plants per replicate, ensuring full methodological reproducibility.

***Comment 2 (Results and Tables):*** *I recommend a final check of units, decimal formatting, statistical notation, and consistency of superscript letters.*

**Response:** We have carefully reviewed all tables. Units, decimal formats, and statistical notations have been standardized, and the consistency of superscript letters has been double-checked to ensure clarity.

***Comment 3 (Discussion):*** *A minor reduction in repetitive statements would make this section more concise and focused.*

**Response:** We have streamlined the Discussion section by removing repetitive statements to improve flow and conciseness while maintaining the focus on physiological interpretation.

***Comment 4 (Conclusion):*** *I suggest a minor language edit to ensure that all statements are fully consistent with the results.*

**Response:** We have carefully reviewed the Conclusion to ensure all statements are precisely aligned with our experimental results, particularly regarding anthocyanin and chlorophyll responses.

***Comment 5 (References and language):*** *A final editorial check is recommended for reference formatting, punctuation, journal names, and consistency.*

**Response:** We have performed a thorough editorial check of the entire manuscript, including the reference list, to ensure consistency in citation style, formatting, and the correction of minor grammatical and typographical errors.

Regards
