# Supplementary material for: Morphophysiological and biochemical responses of two grape cultivars to phosphorus deficiency and toxicity
Source: PLoS One. 2026 Oct 26;21(10):e0361111. doi: 10.1371/journal.pone.0361111 (PMC13704810; doi:10.1371/journal.pone.0361111)
Supplement: Supplementary file 3 [file pone.0361111.s005.docx]

**Dear Handling Editor,**

We thank you and both reviewers for their careful reading of the manuscript and for the constructive suggestions, which have substantially improved the clarity, rigor, and presentation of the work. Below we respond to each comment point by point.

Regards

**Dear In-house Editor,**

We thank you for your suggestions.

**Comment 1:** Journal Requirements: If the reviewer comments include a recommendation to cite specific previously published works, please review and evaluate these publications to determine whether they are relevant and should be cited. There is no requirement to cite these works unless the editor has indicated otherwise. Please review your reference list to ensure that it is complete and correct. If you have cited papers that have been retracted, please include the rationale for doing so in the manuscript text, or remove these references and replace them with relevant current references. Any changes to the reference list should be mentioned in the rebuttal letter that accompanies your revised manuscript. If you need to cite a retracted article, indicate the article’s retracted status in the References list and also include a citation and full reference for the retraction notice.

**Response: Thanks. Noted.**

**Reviewer #1**

We sincerely thank the reviewer for the careful reading of our manuscript and for the constructive comments and suggestions, which have helped us improve the quality and clarity of the paper. We have revised the manuscript thoroughly in response to all concerns raised. Our point-by-point responses are provided below.

Although the authors have revised the manuscript in response to the reviewers’ comments, I do not consider the responses sufficiently complete or convincing to address the major concerns raised during the previous review. In several cases, the authors have mainly modified the wording or added general explanations without providing the additional evidence, clarification, or methodological justification necessary to resolve the underlying concerns.

More importantly, the revision does not convincingly demonstrate that the major scientific concerns identified by the reviewers have been resolved. A revision should not merely respond to individual comments at the textual level; it should provide clear and comprehensive evidence that the fundamental scientific and methodological weaknesses have been addressed. In my assessment, this has not been achieved.
Therefore, I do not believe that another round of minor or textual revision would adequately resolve the remaining problems. Considering the unresolved scientific concerns, the limited strength of the supporting evidence, and the extent to which the conclusions exceed the experimental findings, I do not consider the manuscript suitable for publication in PLOS ONE in its current form. I therefore recommend **rejection**.

**Comment 1: Overly descriptive Discussion & Conclusion / Lack of mechanistic depth**

**Response:** We fully agree with the reviewer’s insightful recommendation. In the revised manuscript, we have overhauled these sections to eliminate redundant data repetition and instead highlight physiological mechanisms, signaling pathways, resource trade-offs, and recent scientific literature.

1. **Refinements in Introduction:**

Minor textual refinements were made to the Introduction to improve contextual clarity and better establish the physiological background of the study.

2. **Revisions in Discussion & Incorporation of New References:**
   - **Integration of New Literature:** We have updated our literature support by adding three relevant and recent publications to reinforce our mechanistic interpretations regarding nutrient transport, gene regulation, and stress adaptation.
   - **Mechanistic Anthocyanin Regulation:** We integrated genetic and biochemical evidence regarding the up-regulation of *VvMYBA1* and *VvUFGT* under Pi starvation to explain anthocyanin accumulation as an energy-diverting defense strategy (citing *Zheng et al., 2020*).
   - **Nutrient Crosstalk (Fe/Zn & P):** We expanded the discussion on systemic signaling networks (PHR1 regulon) and root-to-shoot xylem loading transporters (such as PHO1 and HMA families) to explain the antagonistic suppression of leaf Fe and Zn concentrations under excess P (5 mM), following *Xie et al. (2019)*.
3. **Revisions in Conclusion:**

The Conclusion section was completely synthesized into three cohesive paragraphs focusing on:

- Cultivar-specific vegetative and biomass partitioning across the three P regimes (0 mM, 2.5 mM, and 5 mM).
- Energy trade-offs (growth vs. ROS scavenging/anthocyanin defense) and mineral homeostasis.
- The physiological justification for the controlled 100-day evaluation framework.

**Comment 2: Methodological Timeframe & Experimental Scope**

**Response:** We appreciate this critical observation. We have clarified the rationale behind the 100-day experimental timeline we emphasized that establishing these baseline physiological responses under strictly controlled environmental parameters is an essential prerequisite to isolate direct nutritional effects from complex viability under extreme nutrient stress conditions—specifically complete Pi starvation (0 mM) and toxic excess (5 mM). Furthermore, we emphasized that establishing these baseline physiological responses under strictly controlled environmental parameters is an essential prerequisite to isolate direct nutritional effects from complex soil-climate interactions in open vineyards.

We hope that these revisions adequately address your concerns and improve the quality of our manuscript. We are happy to provide any further clarification if needed.

Regards

**Reviewer #2**

We sincerely thank the reviewer for the careful reading of our manuscript.

**Comment 1:** The manuscript has been carefully revised and the authors have adequately addressed all of the comments and concerns raised during the review process. The methodological, scientific, statistical, and presentation-related issues identified in the previous version have been appropriately addressed, and the revised manuscript is substantially improved in terms of clarity, scientific rigor, and overall organization. The responses provided by the authors are satisfactory, and the corresponding revisions have been appropriately incorporated into the manuscript. The figures, tables, text, and conclusions are now presented in a clear and consistent manner. I did not identify any remaining major scientific or methodological concerns that would prevent publication. Overall, the revised manuscript is well prepared and addresses the issues raised during the review process satisfactorily. I believe that the manuscript is now suitable for publication in its present form.

**Response:** We sincerely thank the reviewer for the careful reading of and accepting our improved manuscript.

Regards
