# Supplementary material for: Morphophysiological and biochemical responses of two grape cultivars to phosphorus deficiency and toxicity
Source: PLoS One. 2026 Oct 26;21(10):e0361111. doi: 10.1371/journal.pone.0361111 (PMC13704810; doi:10.1371/journal.pone.0361111)
Supplement: Peer review history

# Peer review: Decision Letter 0

**DOI:** 10.1371/journal.pone.0361111.r001

**Authors:** Mojtaba Kordrostami

11 Aug 2026

Dear Dr. Khadivi,

Thank you for submitting your manuscript to PLOS ONE. After careful consideration, we feel that it has merit but does not fully meet PLOS ONE’s publication criteria as it currently stands. Therefore, we invite you to submit a revised version of the manuscript that addresses the points raised during the review process.

Please submit your revised manuscript by Oct 10 2026 11:59PM. If you will need more time than this to complete your revisions, please reply to this message or contact the journal office at plosone@plos.org. When you're ready to submit your revision, log on to https://www.editorialmanager.com/pone/ and select the 'Submissions Needing Revision' folder to locate your manuscript file.

- A letter that responds to each point raised by the academic editor and reviewer(s). You should upload this letter as a separate file labeled 'Response to Reviewers'.
- A marked-up copy of your manuscript that highlights changes made to the original version. You should upload this as a separate file labeled 'Revised Manuscript with Track Changes'.
- An unmarked version of your revised paper without tracked changes. You should upload this as a separate file labeled 'Manuscript'.

If you would like to make changes to your financial disclosure, please include your updated statement in your cover letter. Guidelines for resubmitting your figure files are available below the reviewer comments at the end of this letter.

If applicable, we recommend that you deposit your laboratory protocols in protocols.io to enhance the reproducibility of your results. Protocols.io assigns your protocol its own identifier (DOI) so that it can be cited independently in the future. For instructions see: https://journals.plos.org/plosone/s/submission-guidelines#loc-laboratory-protocols. Additionally, PLOS ONE offers an option for publishing peer-reviewed Lab Protocol articles, which describe protocols hosted on protocols.io. Read more information on sharing protocols at https://plos.org/protocols?utm_medium=editorial-email&utm_source=authorletters&utm_campaign=protocols.

As the corresponding author, your ORCID iD is verified in the submission system and will appear in the published article. PLOS supports the use of ORCID, and we encourage all coauthors to register for an ORCID iD and use it as well. Please encourage your coauthors to verify their ORCID iD within the submission system before final acceptance, as unverified ORCID iDs will not appear in the published article. *Only*  the individual author can complete the verification step; PLOS staff *cannot*  verify ORCID iDs on behalf of authors.

We look forward to receiving your revised manuscript.

Kind regards,

Mojtaba Kordrostami, Ph.D.

Academic Editor

PLOS One

Journal Requirements:

When submitting your revision, we need you to address these additional requirements.

1. Please ensure that your manuscript meets PLOS ONE's style requirements, including those for file naming. The PLOS ONE style templates can be found at

https://journals.plos.org/plosone/s/file?id=wjVg/PLOSOne_formatting_sample_main_body.pdf and

https://journals.plos.org/plosone/s/file?id=ba62/PLOSOne_formatting_sample_title_authors_affiliations.pdf.

2. Please include a complete copy of PLOS’ questionnaire on inclusivity in global research in your revised manuscript. Our policy for research in this area aims to improve transparency in the reporting of research performed outside of researchers’ own country or community. The policy applies to researchers who have travelled to a different country to conduct research, research with Indigenous populations or their lands, and research on cultural artefacts. The questionnaire can also be requested at the journal’s discretion for any other submissions, even if these conditions are not met.  Please find more information on the policy and a link to download a blank copy of the questionnaire here: https://journals.plos.org/plosone/s/best-practices-in-research-reporting. Please upload a completed version of your questionnaire as Supporting Information when you resubmit your manuscript.

3. In your Methods section, please provide additional information regarding the permits you obtained for the work. Please ensure you have included the full name of the authority that approved the field site access and, if no permits were required, a brief statement explaining why.

4. In the online submission form you indicate that your data is not available for proprietary reasons and have provided a contact point for accessing this data. Please note that your current contact point is a co-author on this manuscript. According to our Data Policy, the contact point must not be an author on the manuscript and must be an institutional contact, ideally not an individual. Please revise your data statement to a non-author institutional point of contact, such as a data access or ethics committee, and send this to us via return email. Please also include contact information for the third party organization, and please include the full citation of where the data can be found.

5. If the reviewer comments include a recommendation to cite specific previously published works, please review and evaluate these publications to determine whether they are relevant and should be cited. There is no requirement to cite these works unless the editor has indicated otherwise.

[Note: HTML markup is below. Please do not edit.]

Reviewers' comments:

Reviewer's Responses to Questions

**Comments to the Author**

1. Is the manuscript technically sound, and do the data support the conclusions?

Reviewer #1: No

Reviewer #2: Yes

**********

2. Has the statistical analysis been performed appropriately and rigorously?

Reviewer #1: Yes

Reviewer #2: Yes

**********

3. Have the authors made all data underlying the findings in their manuscript fully available?

The PLOS Data policy

Reviewer #1: Yes

Reviewer #2: Yes

**********

4. Is the manuscript presented in an intelligible fashion and written in standard English?

Reviewer #1: No

Reviewer #2: Yes

**********

Reviewer #1: The present article includes a good range of morphological, physiological, nutritional and biochemical indices. However, the article has significant weaknesses in the quality of English writing, clarity of presentation, depth of interpretation and some methodological and reporting issues that make it impossible to accept at present. The article in its current state does not meet the technical and clarity standards of PLOS ONE.

The article's English is full of irregular sentences, grammatical errors, misspellings, and unnatural expressions. Example: "degra70dation"

Some references look suspicious. Check and update all references.

The research gap and the paper's innovation should be more clearly stated.

The discussion is overly descriptive and the in-depth mechanistic interpretation is weak.

The results for enzymes, starch and anthocyanins are interesting but need to be better correlated with grapes.

Limitations of the study (short-term greenhouse experiment, lack of fruit yield and quality studies) should be noted.

Reviewer #2: Comments to the Authors

1. Overall assessment

This is a well-designed and scientifically relevant study addressing an important aspect of grapevine nutrition. The comparative evaluation of ‘Khalili’ and ‘Perlette’ under phosphorus deficiency, sufficiency, and excess provides useful information on genotype-dependent responses to P stress. The integration of morphological, nutritional, antioxidant, and biochemical traits is a particular strength of the manuscript.

2. Introduction

The Introduction is comprehensive and provides a good scientific rationale for the study. The research gap and objectives are clearly presented. Only minor language editing is recommended to improve conciseness and avoid repetition in some paragraphs.

3. Materials and Methods

The experimental design is clearly described, including the 2 × 3 factorial arrangement, completely randomized design, greenhouse conditions, and phosphorus treatments. Minor clarification of the number of plants represented by each replicate would improve reproducibility.

4. Results and Tables

The Results section is generally clear and well organized. The tables provide useful quantitative information and effectively support the main findings. I recommend a final check of units, decimal formatting, statistical notation, and consistency of superscript letters throughout the tables.

5. Discussion

The Discussion is informative and appropriately relates the findings to previous studies. The physiological interpretation of antioxidant enzyme responses and nutrient interactions is particularly interesting. A minor reduction in repetitive statements would make this section more concise and focused.

6. Conclusion

The Conclusion appropriately summarizes the major findings and highlights the differential responses of the two cultivars. I suggest a minor language edit to ensure that all statements are fully consistent with the results presented in the manuscript, particularly regarding anthocyanin and chlorophyll responses.

7. References and language

The reference list is extensive and generally appropriate. A final editorial check is recommended for reference formatting, punctuation, journal names, and consistency of citation style. Some minor grammatical and typographical errors should also be corrected during revision.

**********

what does this mean?). If published, this will include your full peer review and any attached files.

If you choose “no”, your identity will remain anonymous but your review may still be made public.

**Do you want your identity to be public for this peer review?**  For information about this choice, including consent withdrawal, please see our Privacy Policy

Reviewer #1: No

Reviewer #2: No

**********

[NOTE: If reviewer comments were submitted as an attachment file, they will be attached to this email and accessible via the submission site. Please log into your account, locate the manuscript record, and check for the action link "View Attachments". If this link does not appear, there are no attachment files.]

To ensure your figures meet our technical requirements, please review our figure guidelines: https://journals.plos.org/plosone/s/figures

You may also use PLOS’s free figure tool, NAAS, to help you prepare publication quality figures: https://journals.plos.org/plosone/s/figures#loc-tools-for-figure-preparation.

NAAS will assess whether your figures meet our technical requirements by comparing each figure against our figure specifications.

# Author comment: Author response to Decision Letter 1

**DOI:** 10.1371/journal.pone.0361111.r002

24 Aug 2026

Dear Handling Editor,

We thank you and all reviewers for their careful reading of the manuscript and for the constructive suggestions, which have substantially improved the clarity, rigor, and presentation of the work. Below we respond to each comment point by point.

Regards

Response to Reviewer 1

We sincerely thank the reviewer for the careful reading of our manuscript and for the constructive comments and suggestions, which have helped us improve the quality and clarity of the paper. We have revised the manuscript thoroughly in response to all concerns raised. Our point-by-point responses are provided below.

Comment 1: The manuscript contains numerous grammatical errors, spelling mistakes, and unnatural expressions.

Response: We have carefully revised the entire manuscript to improve language quality, grammar, spelling, and style. All typographical errors and unnatural expressions, including specific errors such as “degra70dation,” have been corrected throughout the text.

Comment 2: Some references appear questionable. Please check and update all references.

Response: All references were carefully checked, verified, and updated where necessary to ensure accuracy, consistency, and relevance to the study.

Comment 3: The research gap and novelty should be stated more clearly.

Response: The Introduction section was revised to more clearly delineate the research gap and highlight the novel aspects and significance of this study.

Comment 4: The Discussion section is too descriptive and lacks deep mechanistic interpretation.

Response: The Discussion section was revised to reduce descriptive content and strengthen the mechanistic interpretation of the findings.

Comment 5: The results on enzymes, starch, and anthocyanins are interesting but should be better linked to grape physiology.

Response: We have revised the Discussion to better relate the findings on enzyme activity, starch, and anthocyanins to grape physiology and stress responses.

Comment 6: The limitations of the study should be acknowledged, including the short-term greenhouse experiment and the lack of yield and fruit quality assessments.

Response: We sincerely appreciate the reviewer’s insightful comment. We addressed this point in the revised manuscript by explicitly contextualizing the experimental constraints and the methodological rationale of the study. Specifically, the 100-day experimental duration was governed by plant physiological viability under severe nutrient stress, as prolonging the trial under complete P deficiency (0 mM) and extreme P toxicity (5 mM) would have compromised vine survival and confounded the physiological assays. Furthermore, establishing baseline physiological, morphological, and biochemical indices under strictly controlled greenhouse conditions represented a scientifically indispensable preliminary phase. Given that field environments introduced confounding factors—such as heterogeneous soil matrices, fluctuating moisture regimes, and microclimatic variability—that obscured the direct nutritional effects of the treatments, obtaining robust baseline parameters served as an essential prerequisite to provide a reliable reference framework prior to long-term vineyard evaluations.

Regards

Response to Reviewer 2

We sincerely appreciate your positive assessment of our work and your thoughtful feedback. We are pleased that you found our study design and the assessment of biochemical and morphological traits to be strengths of the manuscript. We have addressed your specific suggestions to improve clarity, precision, and conciseness as follows.

Comment 1 (Materials and Methods): Clarification of the number of plants represented by each replicate would improve reproducibility.

Response: We have revised the Materials and Methods section to explicitly state the number of plants per replicate, ensuring full methodological reproducibility.

Comment 2 (Results and Tables): I recommend a final check of units, decimal formatting, statistical notation, and consistency of superscript letters.

Response: We have carefully reviewed all tables. Units, decimal formats, and statistical notations have been standardized, and the consistency of superscript letters has been double-checked to ensure clarity.

Comment 3 (Discussion): A minor reduction in repetitive statements would make this section more concise and focused.

Response: We have streamlined the Discussion section by removing repetitive statements to improve flow and conciseness while maintaining the focus on physiological interpretation.

Comment 4 (Conclusion): I suggest a minor language edit to ensure that all statements are fully consistent with the results.

Response: We have carefully reviewed the Conclusion to ensure all statements are precisely aligned with our experimental results, particularly regarding anthocyanin and chlorophyll responses.

Comment 5 (References and language): A final editorial check is recommended for reference formatting, punctuation, journal names, and consistency.

Response: We have performed a thorough editorial check of the entire manuscript, including the reference list, to ensure consistency in citation style, formatting, and the correction of minor grammatical and typographical errors.

Regards

Attachment Submitted filename: Response to Reviewers.docx

# Peer review: Decision Letter 1

**DOI:** 10.1371/journal.pone.0361111.r003

**Authors:** Mojtaba Kordrostami

20 Sep 2026

Dear Dr. Khadivi,

Thank you for submitting your manuscript to PLOS One. After careful consideration, we feel that it has merit but does not fully meet PLOS One’s publication criteria as it currently stands. Therefore, we invite you to submit a revised version of the manuscript that addresses the points raised during the review process.

Please submit your revised manuscript by Oct 20 2026 11:59PM. If you will need more time than this to complete your revisions, please reply to this message or contact the journal office at plosone@plos.org. When you're ready to submit your revision, log on to https://www.editorialmanager.com/pone/ and select the 'Submissions Needing Revision' folder to locate your manuscript file.

- A letter that responds to each point raised by the academic editor and reviewer(s). You should upload this letter as a separate file labeled 'Response to Reviewers'.
- A marked-up copy of your manuscript that highlights changes made to the original version. You should upload this as a separate file labeled 'Revised Manuscript with Track Changes'.
- An unmarked version of your revised paper without tracked changes. You should upload this as a separate file labeled 'Manuscript'.

If you would like to make changes to your financial disclosure, please include your updated statement in your cover letter. Guidelines for resubmitting your figure files are available below the reviewer comments at the end of this letter.

If applicable, we recommend that you deposit your laboratory protocols in protocols.io to enhance the reproducibility of your results. Protocols.io assigns your protocol its own identifier (DOI) so that it can be cited independently in the future. For instructions see: https://journals.plos.org/plosone/s/submission-guidelines#loc-laboratory-protocols. Additionally, PLOS One offers an option for publishing peer-reviewed Lab Protocol articles, which describe protocols hosted on protocols.io. Read more information on sharing protocols at https://plos.org/protocols?utm_medium=editorial-email&utm_source=authorletters&utm_campaign=protocols.

As the corresponding author, your ORCID iD is verified in the submission system and will appear in the published article. PLOS supports the use of ORCID, and we encourage all coauthors to register for an ORCID iD and use it as well. Please encourage your coauthors to verify their ORCID iD within the submission system before final acceptance, as unverified ORCID iDs will not appear in the published article. *Only*  the individual author can complete the verification step; PLOS staff *cannot*  verify ORCID iDs on behalf of authors.

We look forward to receiving your revised manuscript.

Kind regards,

Mojtaba Kordrostami, Ph.D.

Academic Editor

PLOS One

Journal Requirements:

If the reviewer comments include a recommendation to cite specific previously published works, please review and evaluate these publications to determine whether they are relevant and should be cited. There is no requirement to cite these works unless the editor has indicated otherwise.

Please review your reference list to ensure that it is complete and correct. If you have cited papers that have been retracted, please include the rationale for doing so in the manuscript text, or remove these references and replace them with relevant current references. Any changes to the reference list should be mentioned in the rebuttal letter that accompanies your revised manuscript. If you need to cite a retracted article, indicate the article’s retracted status in the References list and also include a citation and full reference for the retraction notice.

[Note: HTML markup is below. Please do not edit.]

Reviewers' comments:

Reviewer's Responses to Questions

**Comments to the Author**

Reviewer #1: (No Response)

Reviewer #2: (No Response)

**********

2. Is the manuscript technically sound, and do the data support the conclusions?

Reviewer #1: Partly

Reviewer #2: Yes

**********

3. Has the statistical analysis been performed appropriately and rigorously?

Reviewer #1: Yes

Reviewer #2: Yes

**********

4. Have the authors made all data underlying the findings in their manuscript fully available?

The PLOS Data policy

Reviewer #1: Yes

Reviewer #2: Yes

**********

5. Is the manuscript presented in an intelligible fashion and written in standard English?

Reviewer #1: Yes

Reviewer #2: (No Response)

**********

Reviewer #1: Although the authors have revised the manuscript in response to the reviewers’ comments, I do not consider the responses sufficiently complete or convincing to address the major concerns raised during the previous review. In several cases, the authors have mainly modified the wording or added general explanations without providing the additional evidence, clarification, or methodological justification necessary to resolve the underlying concerns.

More importantly, the revision does not convincingly demonstrate that the major scientific concerns identified by the reviewers have been resolved. A revision should not merely respond to individual comments at the textual level; it should provide clear and comprehensive evidence that the fundamental scientific and methodological weaknesses have been addressed. In my assessment, this has not been achieved.

Therefore, I do not believe that another round of minor or textual revision would adequately resolve the remaining problems. Considering the unresolved scientific concerns, the limited strength of the supporting evidence, and the extent to which the conclusions exceed the experimental findings, I do not consider the manuscript suitable for publication in PLOS ONE in its current form. I therefore recommend **rejection**.

Reviewer #2: The manuscript has been carefully revised and the authors have adequately addressed all of the comments and concerns raised during the review process. The methodological, scientific, statistical, and presentation-related issues identified in the previous version have been appropriately addressed, and the revised manuscript is substantially improved in terms of clarity, scientific rigor, and overall organization.

The responses provided by the authors are satisfactory, and the corresponding revisions have been appropriately incorporated into the manuscript. The figures, tables, text, and conclusions are now presented in a clear and consistent manner. I did not identify any remaining major scientific or methodological concerns that would prevent publication.

Overall, the revised manuscript is well prepared and addresses the issues raised during the review process satisfactorily. I believe that the manuscript is now suitable for publication in its present form.

**********

what does this mean?). If published, this will include your full peer review and any attached files.

If you choose “no”, your identity will remain anonymous but your review may still be made public.

**Do you want your identity to be public for this peer review?**  For information about this choice, including consent withdrawal, please see our Privacy Policy

Reviewer #1: No

Reviewer #2: No

**********

[NOTE: If reviewer comments were submitted as an attachment file, they will be attached to this email and accessible via the submission site. Please log into your account, locate the manuscript record, and check for the action link "View Attachments". If this link does not appear, there are no attachment files.]

To ensure your figures meet our technical requirements, please review our figure guidelines: https://journals.plos.org/plosone/s/figures

You may also use PLOS’s free figure tool, NAAS, to help you prepare publication quality figures: https://journals.plos.org/plosone/s/figures#loc-tools-for-figure-preparation.

NAAS will assess whether your figures meet our technical requirements by comparing each figure against our figure specifications.

# Author comment: Author response to Decision Letter 2

**DOI:** 10.1371/journal.pone.0361111.r004

5 Oct 2026

Dear Handling Editor,

We thank you and both reviewers for their careful reading of the manuscript and for the constructive suggestions, which have substantially improved the clarity, rigor, and presentation of the work. Below we respond to each comment point by point.

Regards

Dear In-house Editor,

We thank you for your suggestions.

Comment 1: Journal Requirements: If the reviewer comments include a recommendation to cite specific previously published works, please review and evaluate these publications to determine whether they are relevant and should be cited. There is no requirement to cite these works unless the editor has indicated otherwise. Please review your reference list to ensure that it is complete and correct. If you have cited papers that have been retracted, please include the rationale for doing so in the manuscript text, or remove these references and replace them with relevant current references. Any changes to the reference list should be mentioned in the rebuttal letter that accompanies your revised manuscript. If you need to cite a retracted article, indicate the article’s retracted status in the References list and also include a citation and full reference for the retraction notice.

Response: Thanks. Noted.

Reviewer #1

We sincerely thank the reviewer for the careful reading of our manuscript and for the constructive comments and suggestions, which have helped us improve the quality and clarity of the paper. We have revised the manuscript thoroughly in response to all concerns raised. Our point-by-point responses are provided below.

Although the authors have revised the manuscript in response to the reviewers’ comments, I do not consider the responses sufficiently complete or convincing to address the major concerns raised during the previous review. In several cases, the authors have mainly modified the wording or added general explanations without providing the additional evidence, clarification, or methodological justification necessary to resolve the underlying concerns.

More importantly, the revision does not convincingly demonstrate that the major scientific concerns identified by the reviewers have been resolved. A revision should not merely respond to individual comments at the textual level; it should provide clear and comprehensive evidence that the fundamental scientific and methodological weaknesses have been addressed. In my assessment, this has not been achieved.

Therefore, I do not believe that another round of minor or textual revision would adequately resolve the remaining problems. Considering the unresolved scientific concerns, the limited strength of the supporting evidence, and the extent to which the conclusions exceed the experimental findings, I do not consider the manuscript suitable for publication in PLOS ONE in its current form. I therefore recommend **rejection**.

Comment 1: Overly descriptive Discussion & Conclusion / Lack of mechanistic depth

Response: We fully agree with the reviewer’s insightful recommendation. In the revised manuscript, we have overhauled these sections to eliminate redundant data repetition and instead highlight physiological mechanisms, signaling pathways, resource trade-offs, and recent scientific literature.

1. Refinements in Introduction:

Minor textual refinements were made to the Introduction to improve contextual clarity and better establish the physiological background of the study.

2. Revisions in Discussion & Incorporation of New References:

o Integration of New Literature: We have updated our literature support by adding three relevant and recent publications to reinforce our mechanistic interpretations regarding nutrient transport, gene regulation, and stress adaptation.

o Mechanistic Anthocyanin Regulation: We integrated genetic and biochemical evidence regarding the up-regulation of VvMYBA1 and VvUFGT under Pi starvation to explain anthocyanin accumulation as an energy-diverting defense strategy (citing Zheng et al., 2020).

o Nutrient Crosstalk (Fe/Zn & P): We expanded the discussion on systemic signaling networks (PHR1 regulon) and root-to-shoot xylem loading transporters (such as PHO1 and HMA families) to explain the antagonistic suppression of leaf Fe and Zn concentrations under excess P (5 mM), following Xie et al. (2019).

3. Revisions in Conclusion:

The Conclusion section was completely synthesized into three cohesive paragraphs focusing on:

o Cultivar-specific vegetative and biomass partitioning across the three P regimes (0 mM, 2.5 mM, and 5 mM).

o Energy trade-offs (growth vs. ROS scavenging/anthocyanin defense) and mineral homeostasis.

o The physiological justification for the controlled 100-day evaluation framework.

Comment 2: Methodological Timeframe & Experimental Scope

Response: We appreciate this critical observation. We have clarified the rationale behind the 100-day experimental timeline we emphasized that establishing these baseline physiological responses under strictly controlled environmental parameters is an essential prerequisite to isolate direct nutritional effects from complex viability under extreme nutrient stress conditions—specifically complete Pi starvation (0 mM) and toxic excess (5 mM). Furthermore, we emphasized that establishing these baseline physiological responses under strictly controlled environmental parameters is an essential prerequisite to isolate direct nutritional effects from complex soil-climate interactions in open vineyards.

We hope that these revisions adequately address your concerns and improve the quality of our manuscript. We are happy to provide any further clarification if needed.

Regards

Reviewer #2

We sincerely thank the reviewer for the careful reading of our manuscript.

Comment 1: The manuscript has been carefully revised and the authors have adequately addressed all of the comments and concerns raised during the review process. The methodological, scientific, statistical, and presentation-related issues identified in the previous version have been appropriately addressed, and the revised manuscript is substantially improved in terms of clarity, scientific rigor, and overall organization. The responses provided by the authors are satisfactory, and the corresponding revisions have been appropriately incorporated into the manuscript. The figures, tables, text, and conclusions are now presented in a clear and consistent manner. I did not identify any remaining major scientific or methodological concerns that would prevent publication. Overall, the revised manuscript is well prepared and addresses the issues raised during the review process satisfactorily. I believe that the manuscript is now suitable for publication in its present form.

Response: We sincerely thank the reviewer for the careful reading of and accepting our improved manuscript.

Regards

Attachment Submitted filename: Response_to_Reviewers_auresp_2.docx

# Editor report: Decision Letter 2

**DOI:** 10.1371/journal.pone.0361111.r005

**Authors:** Mojtaba Kordrostami

7 Oct 2026

Morphophysiological and biochemical responses of two grape cultivars to phosphorus deficiency and toxicity

PONE-D-26-29407R2

Dear Dr. Khadivi,

We’re pleased to inform you that your manuscript has been judged scientifically suitable for publication and will be formally accepted for publication once it meets all outstanding technical requirements.

Within one week, you’ll receive an e-mail detailing the required amendments. When these have been addressed, you’ll receive a formal acceptance letter and your manuscript will be scheduled for publication.

An invoice will be generated when your article is formally accepted. Please note, if your institution has a publishing partnership with PLOS and your article meets the relevant criteria, all or part of your publication costs will be covered. Please make sure your user information is up-to-date by logging into Editorial Manager at Editorial Manager® and clicking the ‘Update My Information' link at the top of the page. For questions related to billing, please contact billing support.

If your institution or institutions have a press office, please notify them about your upcoming paper to help maximize its impact. If they’ll be preparing press materials, please inform our press team as soon as possible -- no later than 48 hours after receiving the formal acceptance. Your manuscript will remain under strict press embargo until 2 pm Eastern Time on the date of publication. For more information, please contact onepress@plos.org.

Kind regards,

Mojtaba Kordrostami, Ph.D.

Academic Editor

PLOS One

Additional Editor Comments (optional):

Thanks for revising the manuscript carefully.

The manuscript can be accepted now.

Regards

M. Kordrostami, Ph.D.,

Reviewers' comments:

# Editor report: Acceptance letter

**DOI:** 10.1371/journal.pone.0361111.r006

**Authors:** Mojtaba Kordrostami

PONE-D-26-29407R2

PLOS One

Dear Dr. Khadivi,

I'm pleased to inform you that your manuscript has been deemed suitable for publication in PLOS One. Congratulations! Your manuscript is now being handed over to our production team.

At this stage, our production department will prepare your paper for publication. This includes ensuring the following:

* All references, tables, and figures are properly cited

* All relevant supporting information is included in the manuscript submission,

* There are no issues that prevent the paper from being properly typeset

You will receive further instructions from the production team, including instructions on how to review your proof when it is ready. Please keep in mind that we are working through a large volume of accepted articles, so please give us a few days to review your paper and let you know the next and final steps.

Lastly, if your institution or institutions have a press office, please let them know about your upcoming paper now to help maximize its impact. If they'll be preparing press materials, please inform our press team within the next 48 hours. Your manuscript will remain under strict press embargo until 2 pm Eastern Time on the date of publication. For more information, please contact onepress@plos.org.

You will receive an invoice from PLOS for your publication fee after your manuscript has reached the completed accept phase. If you receive an email requesting payment before acceptance or for any other service, this may be a phishing scheme. Learn how to identify phishing emails and protect your accounts at https://explore.plos.org/phishing.

If we can help with anything else, please email us at customercare@plos.org.

Thank you for submitting your work to PLOS One and supporting open access.

Kind regards,

PLOS One Editorial Office Staff

on behalf of

Dr. Mojtaba Kordrostami

Academic Editor

PLOS One
